# Heterocellular Contacts with Mouse Brain Endothelial Cells *Via* Laminin and α6β1 Integrin Sustain Subventricular Zone (SVZ) Stem/Progenitor Cells Properties

**DOI:** 10.3389/fncel.2016.00284

**Published:** 2016-12-15

**Authors:** Alexandra I. Rosa, Sofia Grade, Sofia D. Santos, Liliana Bernardino, Thomas C. Chen, João Relvas, Florence M. Hofman, Fabienne Agasse

**Affiliations:** ^1^Centre for Neuroscience and Cell Biology, University of CoimbraCoimbra, Portugal; ^2^Department of Pathology, University of Southern CaliforniaLos Angeles, CA, USA; ^3^Department of Neurological Surgery, University of Southern CaliforniaLos Angeles, CA, USA; ^4^Glial Cell Biology Group, Institute for Molecular and Cell Biology – IBMCPorto, Portugal; ^5^Instituto de Investigação e Inovação em Saúde, Universidade do PortoPorto, Portugal; ^6^Health Sciences Research Centre, Faculty of Health Sciences, University of Beira InteriorCovilhã, Portugal

**Keywords:** SVZ, neurogenesis, α6β1 integrin, laminin, stemness

## Abstract

Neurogenesis in the subventricular zone (SVZ) is regulated by diffusible factors and cell–cell contacts. *In vivo*, SVZ stem cells are associated with the abluminal surface of blood vessels and such interactions are thought to regulate their neurogenic capacity. SVZ neural stem cells (NSCs) have been described to contact endothelial-derived laminin *via* α6β1 integrin. To elucidate whether heterocellular contacts with brain endothelial cells (BEC) regulate SVZ cells neurogenic capacities, cocultures of SVZ neurospheres and primary BEC, both obtained from C57BL/6 mice, were performed. The involvement of laminin-integrin interactions in SVZ homeostasis was tested in three ways. Firstly, SVZ cells were analyzed following incubation of BEC with the protein synthesis inhibitor cycloheximide (CHX) prior to coculture, a treatment expected to decrease membrane proteins. Secondly, SVZ cells were cocultured with BEC in the presence of an anti-α6 integrin neutralizing antibody. Thirdly, BEC were cultured with β1^−/−^ SVZ cells. We showed that contact with BEC supports, at least in part, proliferation and stemness of SVZ cells, as evaluated by the number of BrdU positive (+) and Sox2+ cells in contact with BEC. These effects are dependent on BEC-derived laminin binding to α6β1 integrin and are decreased in cocultures incubated with anti-α6 integrin neutralizing antibody and in cocultures with SVZ β1^−/−^ cells. Moreover, BEC-derived laminin sustains stemness in SVZ cell cultures *via* activation of the Notch and mTOR signaling pathways. Our results show that BEC/SVZ interactions involving α6β1 integrin binding to laminin, contribute to SVZ cell proliferation and stemness.

## Introduction

Stem cells of the rodent subventricular zone (SVZ) reside in a specific microenvironment: the neurogenic niche, which contributes to the maintenance of their intrinsic capacities. Identification of the cellular and molecular components of this niche is fundamental to understanding how stemness is maintained and may provide crucial targets for the expansion of stem cells for therapeutic purposes.

SVZ neural stem cells (NSCs), identified as a subset of astrocytes, called B cells, lie in the wall of the lateral ventricles (Ming and Song, 2011). The immediate progeny of NSCs, the transient-amplifying progenitors (C cells) give rise mainly to neuroblasts (A cells) that migrate tangentially toward the olfactory bulb (Zhao et al., 2008). Numerous diffusible and cell contact factors modulate stem cell maintenance, proliferation, neuronal differentiation and migration and are provided by cellular components constituting the niche (Moyse et al., 2008; Coronas, 2009).

The vasculature plays a central role in stem cell regulation (Goldberg and Hirschi, 2009; Koutsakis and Kazanis, 2016). It has been shown that the density of the vascular network is higher in the periventricular striatal wall, i.e., the most neurogenic part of the SVZ (Kazanis et al., 2010). Stem/progenitor cells in the SVZ are found associated with the abluminal surface of blood vessels (Capela and Temple, 2002). Proximity to blood vessels is a general feature of stem cell niches. Indeed, cancer stem cells in brain tumors, undifferentiated spermatogonia in the testis and haematopoietic stem cells in the bone marrow closely associate with the vasculature (Calabrese et al., 2007; Yoshida et al., 2007; Coskun and Hirschi, 2010). In the hippocampus, the other main neurogenic area of the brain, radial glia-like stem cells of the dentate gyrus (DG) extend processes toward the molecular layer to wrap blood vessels (Moss et al., 2016). Endothelial cells (EC) secrete diffusible factors that direct stem/progenitor cell fate and proliferation (Shen et al., 2004; Plane et al., 2010; Crouch et al., 2015). These factors, including angiopoietins, betacellulin (BTC), brain-derived neurotrophic factor (BDNF), vascular endothelial growth factor (VEGF), neurotrophin-3 (NT3), placental growth factor 2 (PlGF-2), fibroblast growth factor-2 (FGF-2) and pigment epithelium-derived growth factor (PEDF) modulate stem cell dynamics as a result of paracrine diffusion directly from EC (Louissaint et al., 2002; Mercier et al., 2002; Ohab et al., 2006; Ramírez-Castillejo et al., 2006; Rosa et al., 2010; Sun et al., 2010; Gómez-Gaviro et al., 2012; Delgado et al., 2014; Crouch et al., 2015) or *via* fractones, structures from the extracellular matrix (ECM) that extend from EC, sequester EC-derived factors and contact NSCs (Kerever et al., 2007). Stem/progenitor cells contact EC directly in patches of vessels lacking astrocytes endfeet and pericyte coverage (Tavazoie et al., 2008). These contacts support proliferation and self-renewal in tumor cells *via* activation of the Notch signaling pathway (Hovinga et al., 2010; Zhu et al., 2011). In the SVZ, adhesion of B and C cells to vessels is dependent on the expression of transmembrane α6β1 integrin that binds EC-derived ECM laminin (Shen et al., 2008; Kokovay et al., 2010). Whether these cell–cell contacts directly sustain proliferation and self-renewal remains to be shown.

The present work was undertaken to identify the relationship between SVZ stem cells and EC. Using cocultures of SVZ neurospheres with primary brain endothelial cells (BEC), we found that binding of SVZ *via* α6β1 integrin to laminin-rich ECM holds stem cell maintenance.

## Materials and methods

The experimental protocol was designed taking into account the Russel and Burch 3R's principle and was approved by the Institutional and the Portuguese General Veterinary Board Ethical Committees in accordance with the National and European Union rules. Part of the experiments were performed in USC after the approval of animal protocols by the USC Institutional Animal Care and Use Committee.

### Cell cultures

SVZ neurospheres were prepared from 1- to 3-day-old C57BL/6 WT or GFP mice in serum-free medium (SFM) supplemented with 10 ng/ml epidermal growth factor (EGF) and 5 ng/ml FGF-2 (Invitrogen) (Agasse et al., 2008). BEC were obtained from adult (6–8 weeks) mice whole brain fragments (excluding the brain stem and the cerebellum) digested with 1 mg/ml of collagenase/dispase (Roche) and resuspended in EC medium containing 10% of fetal bovine serum (FBS) (Wu et al., 2003). BEC were selected using 4 μg/ml puromycin for 2 days (Perrière et al., 2005). Cells were plated on 1% gelatin A (Sigma-Aldrich)-coated petri-dishes, grown until confluence (10 days), trypsinized and collected. BEC looked healthier and maintained better as subconfluent cultures, compared to confluent cultures. This was especially evident at higher passages. At increased density of BEC, the cells were more quiescent, and eventually lifted off the substrate. Thus, BEC were grown to confluency only for expansion purposes. In cocultures, we used BEC at no more than 60% confluency.

For cocultures, BEC were plated on gelatin-coated glass coverslips in 24-well plates (20,000 cells/well), in EC medium for 24 h, treated with or without (Control) the protein synthesis inhibitor cycloheximide (CHX; 1 μg/ml; Sigma-Aldrich) for 1 h and carefully washed 3 times in sterile PBS to completely remove traces of FBS and/or CHX. SVZ spheres were seeded on top of BEC in SFM devoid of growth factors. The contribution of BEC soluble factors was evaluated in SVZ neurospheres plated on CHX-treated BEC in SFM plus BEC SFM-conditioned medium (CM) (1:1). After 24 h, cells were fixed in 4% paraformaldehyde. For cell proliferation studies, 10 μM 5-bromo-2′-deoxyuridine (BrdU; Sigma-Aldrich) was added to the medium for the last 4 h of coculture session.

For Western blot (see Western blot section), SVZ cells were obtained from the dissociation of primary neurospheres and plated as single cells on ECM proteins to allow a homogeneous activation of stemness and the Notch pathway rather than a selective activation affecting only cells present at the bottom of the neurospheres contacting the substrate. For the Cell pair assay (see Cell pair assay section), SVZ cells were obtained from the dissociation of SVZ fragments and plated as single cells on ECM proteins, to allow the testing of SVZ cells that had not been previously exposed to growth factors.

### Genetic ablation of β1 in neurospheres

SVZ neurospheres obtained from floxed β1 mice (β1^flox/flox^) (1- to 4-day) were grown in DMEM/F12 supplemented with B27 in the presence of EGF (10 ng/ml) and FGF-2 (5 ng/ml) for 3 days, then dissociated and infected with an adenoviral vector expressing Cre recombinase (Eton Bioscience Inc.) using 50 virus particles per cell (Leone et al., 2005). Cells were replated in the same medium. The culture medium was changed after 3 days to medium without adenovirus. Recombination was confirmed 10 days after infection by the expression of β-galactosidase in the primary infected neurospheres, as excision of the β1 gene activates a lacZ reporter gene.

### Immunocytochemistry

The terminal deoxynucleotidyl transferase dUTP nick end labeling (TUNEL, Roche) method was used to stain apoptotic nuclei (Rosa et al., 2010). BrdU immunostaining was performed as described previously (Rosa et al., 2010). Cells were incubated overnight with primary antibodies as listed in Supplementary Table 1, and for 1 h with the appropriate Alexa 594 and 488 secondary antibodies (1:200 in PBS, Invitrogen).

SVZ neurospheres and BEC were bound to slides using cytocentrifugation, and labeled for α6 integrin and laminin as aforementioned. SVZ labeling for β1 integrin was performed in living cells incubated at 37°C for 3 h in SFM containing anti-β1 antibody before fixation. BEC in cocultures were stained for cluster of differentiation 31 (CD31). Nuclei were stained with 2 μg/ml Hoechst 33342 (Invitrogen) and preparations were mounted in Dako mounting medium (Dako).

### Western blot analysis

Detection of α6β1 integrin and laminin was performed in SVZ primary neurospheres and BEC collected in lysis buffer as previously described (Rosa et al., 2010). Total brain proteins were used as a positive control for α6 and β1 integrins. Activation of the Notch pathway was evaluated in SVZ cells obtained from the dissociation of primary neurospheres (Neurocult chemical dissociation kit, STEMCELL Technologies) and plated, as single cells, at a density of 900 000 cells per well of a 6-well plate coated with poly-D-lysine (20 μg/ml) alone or coated with either laminin-1 (25 μg/ml, reference: L2020), fibronectin (5 μg/ml) or vitronectin (5 μg/ml) (all from Sigma-Aldrich). Cells were grown for 72 h in SFM and harvested in lysis buffer. Western blot was performed as previously detailed (Rosa et al., 2010) using primary and secondary antibodies as listed in Supplementary Tables 2, 3, followed by visualization using ECF™ reagent on a Storm 860 Gel and Blot Imaging System (GE Healthcare) or using fluorescence-conjugated secondary antibodies on an Odyssey® Infrared Imaging System (Licor Biosciences). Band intensities were measured using ImageJ (NIH Image).

### Cell pair assay

Dissociated cells from SVZ explants were plated onto 10 mm diameter glass coverslips coated with poly-D-lysine (20 μg/ml) alone or with laminin-1, fibronectin or vitronectin as aforementioned, at a density of 2500 cells per coverslip. Cells were grown in SFM containing 5 ng/ml EGF and 2.5 ng/ml FGF-2 (low EGF/FGF-2) for 24 h. Involvement of the mTOR signaling pathway was tested by incubating cells for the 24 h of the assay in the presence of 20 nM rapamycin (Tocris Bioscience). Cells were immunostained for Sox2 and stained with Hoechst 33342.

### Data analysis

Fluorescent images were acquired using a LSM 510 Meta confocal microscope or an Axioskop 2 Plus fluorescent microscope (Carl Zeiss Inc.). In cocultures, 10 photos (40x magnification) of each coverslip were taken using a LSM 510 Meta confocal microscope (Carl Zeiss). Countings were performed in the vicinity of neurospheres where SVZ cells migrate out of the neurosphere to form a pseudomonolayer of cells. Some of these SVZ cells associate closely with BEC. Comparisons regarding cell proliferation, cell death, stemness, and neuronal differentiation were made between the population of cells that contacts BEC vs. the one that does not contact BEC. Due to the high density of cells, no countings are performed within neurospheres. The percentages of BrdU+/TUNEL+/Sox2+/Mash1+ SVZ cells were therefore calculated within the population of SVZ cells contacting CD31+ BEC (i.e., Hoechst-labeled SVZ cell nuclei that are located <10 μm from BEC) as well as in areas where no BEC are present, for comparison. Numbers of DCX+ ramifications contacting or crossing BEC as well as DCX+ cell bodies adjacent to BEC were counted. Numbers of Sox2+/Sox2+, Sox2+/Sox2− and Sox2−/Sox2− cell pairs were expressed as a percentage of total cell divisions. Unless otherwise specified, experiments were replicated at least in 3 independent cultures. Within each experiment, 3 coverslips for each condition were analyzed. In Western blots, the ratio of intensity between the bands and their respective loading controls (β-actin or GAPDH) were performed. The Western blots presented are representative of blots performed with at least 3 different cultures. Data are expressed as means ± s.e.m. The unpaired Student *t*-test, and the one-way ANOVA followed by the Dunnett post-test for comparison with the control condition or the Bonferroni for multiple comparisons were used; *P* ≤ 0.05 was considered statistically significant.

## Results

### SVZ cells spread on BEC monolayers in cocultures

**Figure 1:**
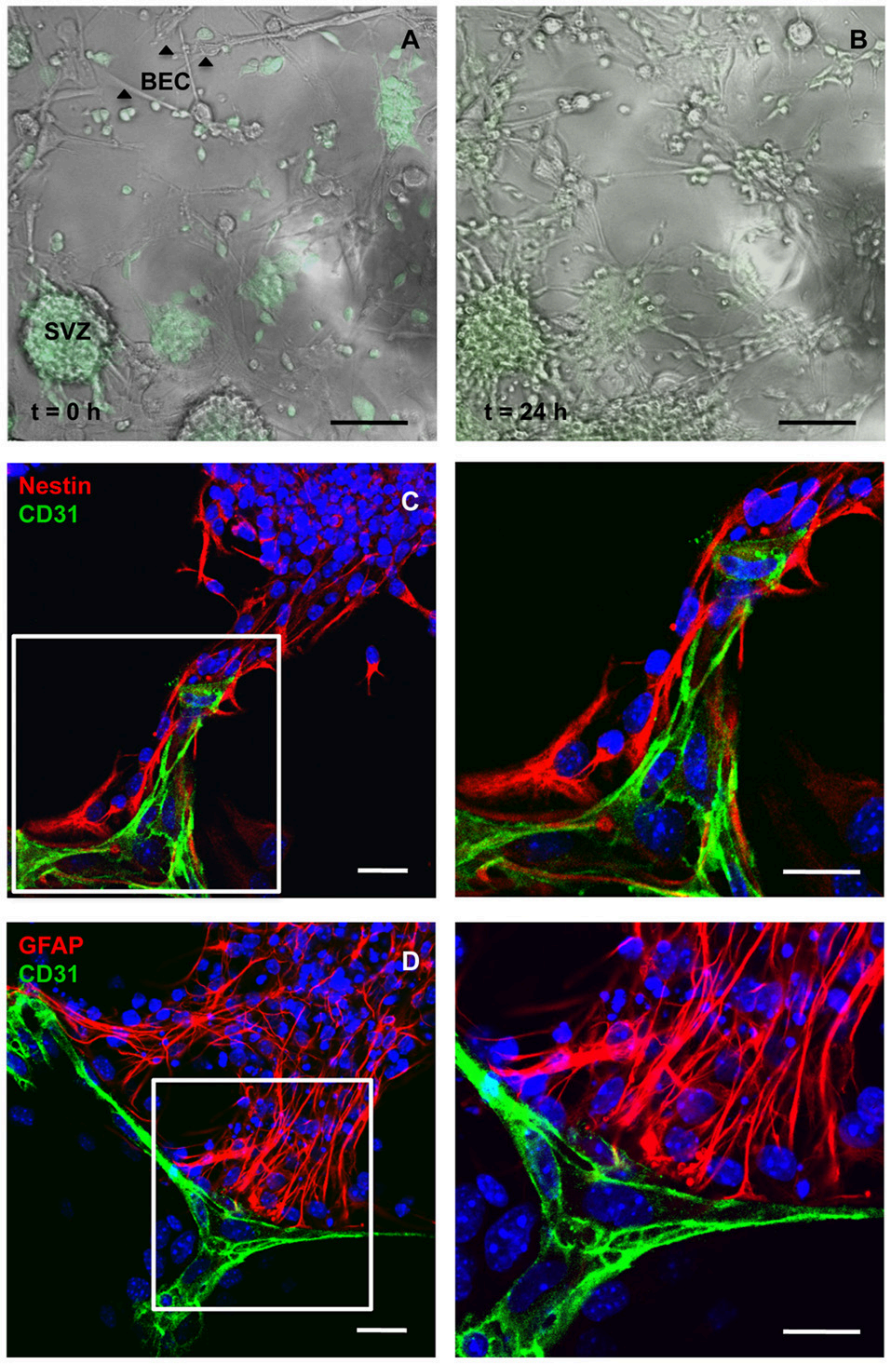
**Cocultures of SVZ cells and BEC**. Representative transmission and fluorescence digital images of a BEC and GFP-expressing SVZ neurospheres coculture at the beginning of the coculture **(A)** and after 24 h **(B)**. Scale bars, 100 μm. **(C,D)** Confocal digital images of a 24 h coculture of CD31+ BEC (green) and SVZ cells showing Nestin+ immature cells (red, **C**) and GFAP+ astrocytes (red, **D**) contacting BEC. Nuclei are stained with Hoechst 33342 in blue. Fluorescent images at the right are magnifications of fields shown in the left figures. Scale bars, 20 μm.

BEC primary cultures were grown in direct contact with SVZ cells for 24 h. The maximum duration of the coculture experiment was determined according to the capacity of BEC to survive in SFM and SFM conditioned by SVZ cells (evaluated by Methylthiazol Tetrazolium Assay and TUNEL staining; Supplementary Figure 1). Figure 1 provides images of the interaction between BEC and SVZ cells, demonstrating GFP neurospheres adhering to BEC and extending a pseudomonolayer of cells (Figure 1A, at *t* = 0 h, and Figure 1B, at *t* = 24 h). After 24 h of coculture, SVZ cells including Nestin positive (+) immature cells and GFAP+ astrocytes contact CD31+ BEC (Figures 1C,D).

### Laminin and α6β1 integrin expression in BEC and SVZ

**Figure 2:**
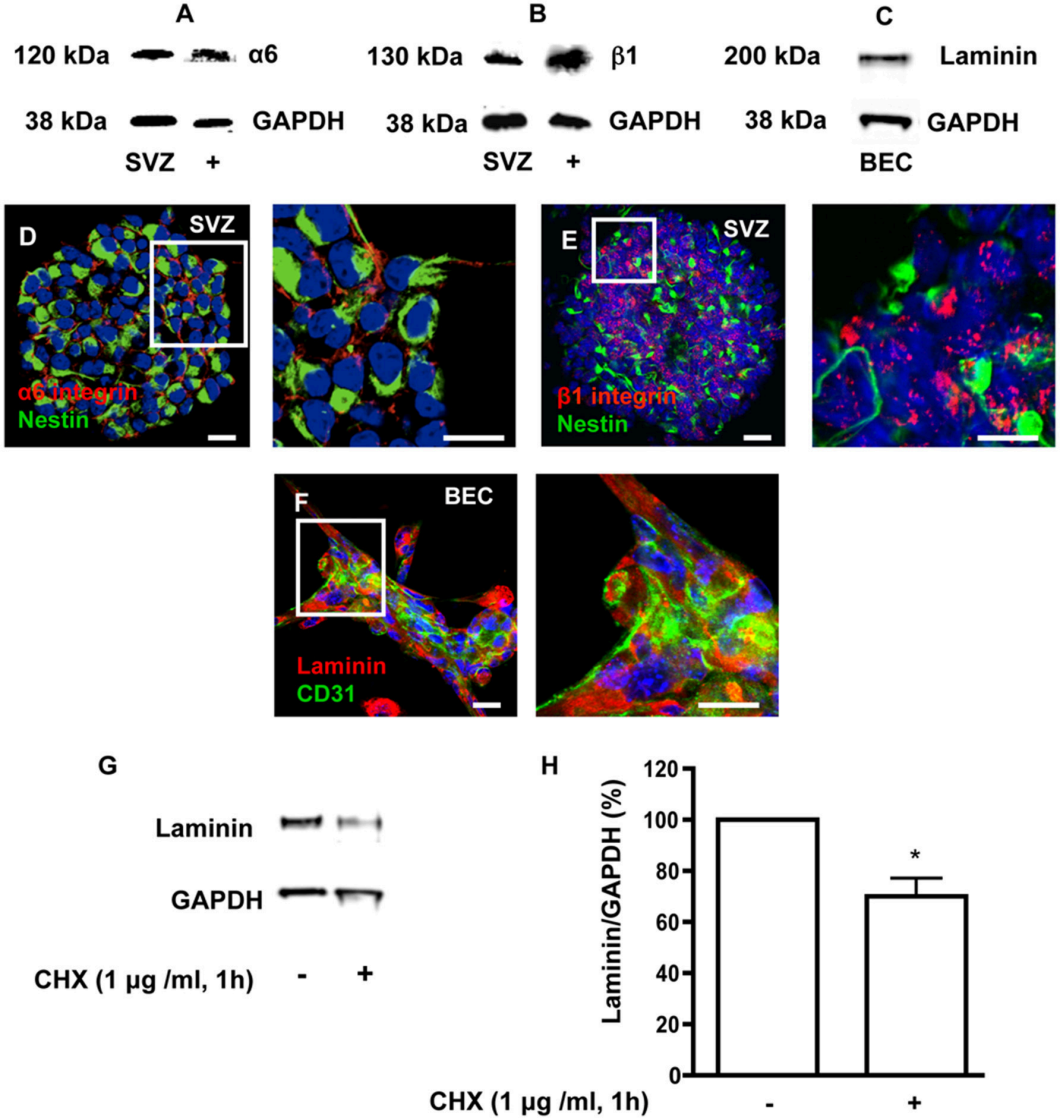
**SVZ cells express α6 and β1 integrins and BEC express laminin. (A,B)** Detection of α6 **(A)** and β1 **(B)** integrin proteins by Western blotting in SVZ neurospheres and in whole brain extracts (positive control). **(C)** Detection of laminin by Western blotting in BEC. GAPDH protein detection was used as a loading control. **(D,E)** Representative confocal digital images depicting the presence of α6 **(D)** and β1 **(E)** integrin subunits in SVZ neurospheres (red staining for α6 and β1 integrins and green staining for Nestin, Hoechst 33342 nuclear staining in blue). Scale bars, 20 μm. **(F)** Confocal digital image shows the expression of laminin in BEC (green staining for CD31, red staining for laminin, Hoechst 33342 nuclear staining in blue). Scale bars, 20 μm. Cycloheximide (CHX) downregulates laminin in BEC. **(G)** Western blot showing laminin protein levels in untreated BEC cultures and in BEC treated with 1 μg/ml CHX for 1 h. GAPDH protein detection was used as a loading control. **(H)** Bar graphs show the respective quantification of laminin levels. ^*^P < 0.05, using the unpaired Student *t*-test for comparison with untreated BEC.

Heterocellular contacts between EC and stem/progenitor cells or cancer stem cells involve binding of laminin to α6β1 integrin (Shen et al., 2008; Lathia et al., 2010). As depicted in Figure 2, free-floating SVZ spheres express α6 and β1 integrin subunits (Figures 2A,B,D,E) and BEC secrete laminin (Figures 2C,F). In order to impair laminin-integrin interactions and test functional implications of these interactions, a pulse of 1 h with 1 μg/ml of the protein synthesis inhibitor cycloheximide (CHX) was applied to BEC followed by a chase of 24 h in SFM to mimic coculture conditions. This treatment was expected to decrease the turnover of ECM and membrane-bound proteins. Laminin protein levels, evaluated by Western blot (Figure 2G) and expressed as a percentage of GAPDH expression (Figure 2H), showed a marked decrease in CHX-treated BEC as compared to untreated cocultures (Untreated BEC vs. CHX-treated BEC: *P* < 0.05). The CHX treatment did not induce apoptosis in BEC as evaluated by TUNEL staining and compared to BEC cultured in EC medium (Control) (Control 24 h: 1.50 ± 0.33%, 3182 cells counted; CHX-treated: 2.54 ± 0.36%, 2271 cells counted).

### Contact with BEC *via* laminin binding to α6β1 integrin promotes proliferation of SVZ cells without affecting cell survival

**Figure 3:**
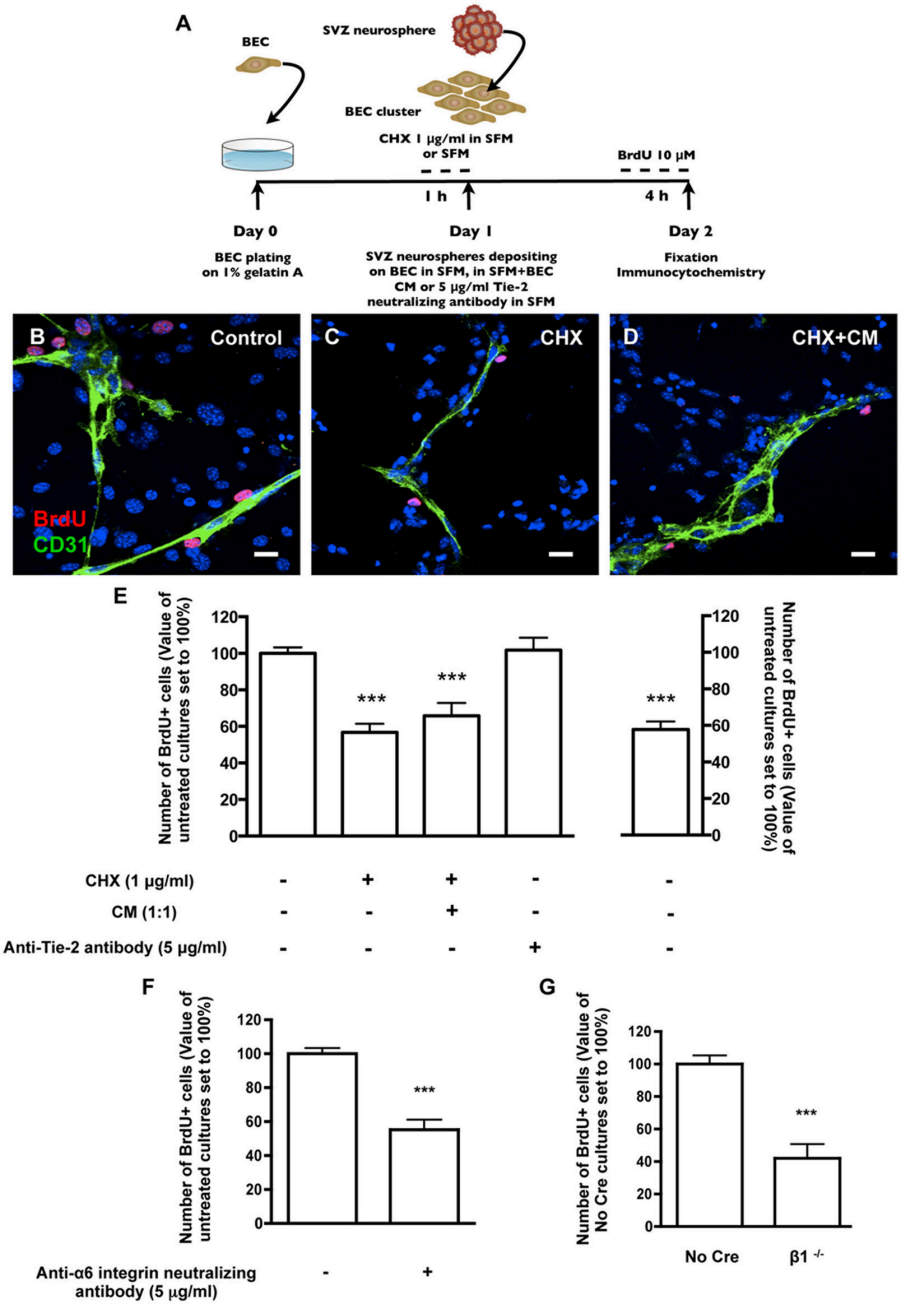
**Heterocellular contacts with BEC promote SVZ cell proliferation. (A)** Experimental protocol. **(B–D)** Representative confocal digital images depicting proliferating cells in BEC and SVZ cells cocultures (green staining for CD31, red staining for BrdU and Hoechst 33342 staining in blue). **(B)** Control cocultures. **(C)** Cocultures with CHX-treated BEC, in SFM. **(D)** Cocultures with CHX-treated BEC, in SFM and BEC-CM (1:1). Scale bars, 20 μm. **(E)** Left: Bar graphs show the number of BrdU+ cells as a percentage of the total cells contacting BEC. Right: Graph displays the number of proliferating cells as a percentage of the total number of cells in the pseudomonolayer of SVZ cells, i.e., non-contacting BEC. Control values were normalized to 100%. ^***^P < 0.001, using one-way ANOVA. **(F,G)** α6β1 integrin mediates proliferation induced by contact with BEC. Bar graph shows the number of BrdU+ cells as a percentage of the total cells contacting BEC in Control cocultures, in anti-α6 integrin neutralizing antibody-incubated cocultures **(F)** and in cocultures with β1^−/−^ SVZ cells **(G)**. ^***^P < 0.001 using the unpaired *t*-test for comparison to Control or No Cre cocultures.

The effect of SVZ-BEC contact on SVZ cell proliferation was evaluated in cocultures incubated with 10 μM BrdU (Figure 3A). Within the population of cells contacting CD31+ BEC (1533 Hoechst-labeled SVZ cells in contact with BEC were counted), 17.08 ± 1.22% were BrdU+. This number was normalized to 100% (Figures 3B,E). In contrast, in pseudomonolayers of SVZ cells that did not contact BEC, fewer cells were proliferating (*P* < 0.001, 4377 Hoechst-labeled SVZ cells not contacting BEC were counted, Figure 3E) indicating that the contact with BEC sustains SVZ cell proliferation. To impair normal SVZ-BEC contacts and evaluate the impact on proliferation of SVZ cells, BEC were cultured for 1 h with 1 μg/ml CHX prior to coculture (Figure 3A). The number of proliferating SVZ cells associated with CHX-treated BEC drastically decreased (*P* < 0.001, Figures 3C,E). As demonstrated above, CHX treatment leads to a decline of laminin protein expression to ~70% of control levels. However, since CHX does not specifically inhibit the synthesis of membrane-bound and ECM proteins as laminin, the observed decrease in proliferation could be due to a reduction in BEC-secreted soluble factors with a proliferative effect on SVZ cells. Nonetheless, in cocultures with CHX-treated BEC where BEC-derived soluble factors were restored by incubation in BEC conditioned media (Figure 3D), the pro-proliferative effects were not recovered (*P* < 0.001, Figure 3E). We have previously shown that the BEC diffusible factor angiopoietin 1 (Ang-1) promotes proliferation in SVZ cells (Rosa et al., 2010). Also, accumulation of diffusible molecules in the ECM may influence progenitor cell dynamics (Kerever et al., 2007). BEC-secreted Ang-1 may therefore accumulate in the ECM and stimulate proliferation. To test this, cocultures were performed in the presence of 5 μg/ml of an anti-Tie2 receptor neutralizing antibody. No difference in the numbers of proliferating SVZ cells contacting BEC was observed (Figure 3E) suggesting that proliferation was mediated by cell contact rather than by Ang-1.

Regarding survival, there were no differences in the numbers of apoptotic TUNEL+ cells between the population of SVZ cells contacting BEC (15.74 ± 1.72% apoptotic nuclei, 1023 cells counted) and the population of SVZ cells not contacting BEC (15.67 ± 2.51% apoptotic nuclei, 4330 cells counted). Moreover, BEC pre-treatment with CHX did not affect the number of apoptotic SVZ cells in contact with BEC (19.34 ± 2.48% apoptotic nuclei, 735 cells counted). These results indicate that SVZ-BEC interactions promote SVZ cell proliferation without interfering with cell survival.

To specifically target cell contacts through α6β1 integrin, cocultures were performed in the presence of an anti-α6 neutralizing antibody (5 μg/ml). We verified by TUNEL staining that the neutralizing antibody did not affect cell death in the population of SVZ cells contacting BEC (13.25 ± 1.86% apoptotic nuclei, 679 cells counted) nor in the pseudomonolayer (14.90 ± 1.52%, 2572 cells counted), as compared to cocultures performed in the absence of the antibody. Regarding proliferation, in the presence of the antibody, the number of BrdU+ cells contacting BEC decreased (*P* < 0.001, Figure 3F) compared with control cocultures. To further confirm this, SVZ neurospheres were obtained from neonatal mice containing floxed β1 alleles (Campos et al., 2004). When exposed to an adenovirus carrying Cre recombinase, SVZ neurospheres with the floxed β1 genetic background lost their capacity to express β1 integrin (β1^flox/flox^ treated with Cre referred to as “β1^−/−^”) (Leone et al., 2005). Control neurospheres expressed β1 integrin (β1^flox/flox^ not treated with Cre, referred to as “No Cre”). No Cre and β1^−/−^ neurospheres were cocultured for 24 h with BEC. The numbers of No Cre BrdU+ cells contacting BEC were similar to that obtained with WT SVZ cells (20.25 ± 5.86%, 893 cells counted). This value was normalized to 100%. In line with the results using anti-α6 neutralizing reagent, the percentage of β1^−/−^ BrdU+ cells in contact with BEC was decreased (*P* < 0.001, Figure 3G). We verified that these effects were not due to a decrease in the proliferative capacities of β1^−/−^ cultures compared to No Cre (data not shown). These data indicate that α6β1 integrin-mediated signaling is responsible, at least in part, for the proliferation of SVZ cells.

### Contact with BEC *via* laminin binding to α6β1 integrin sustains SVZ cell stemness

**Figure 4:**
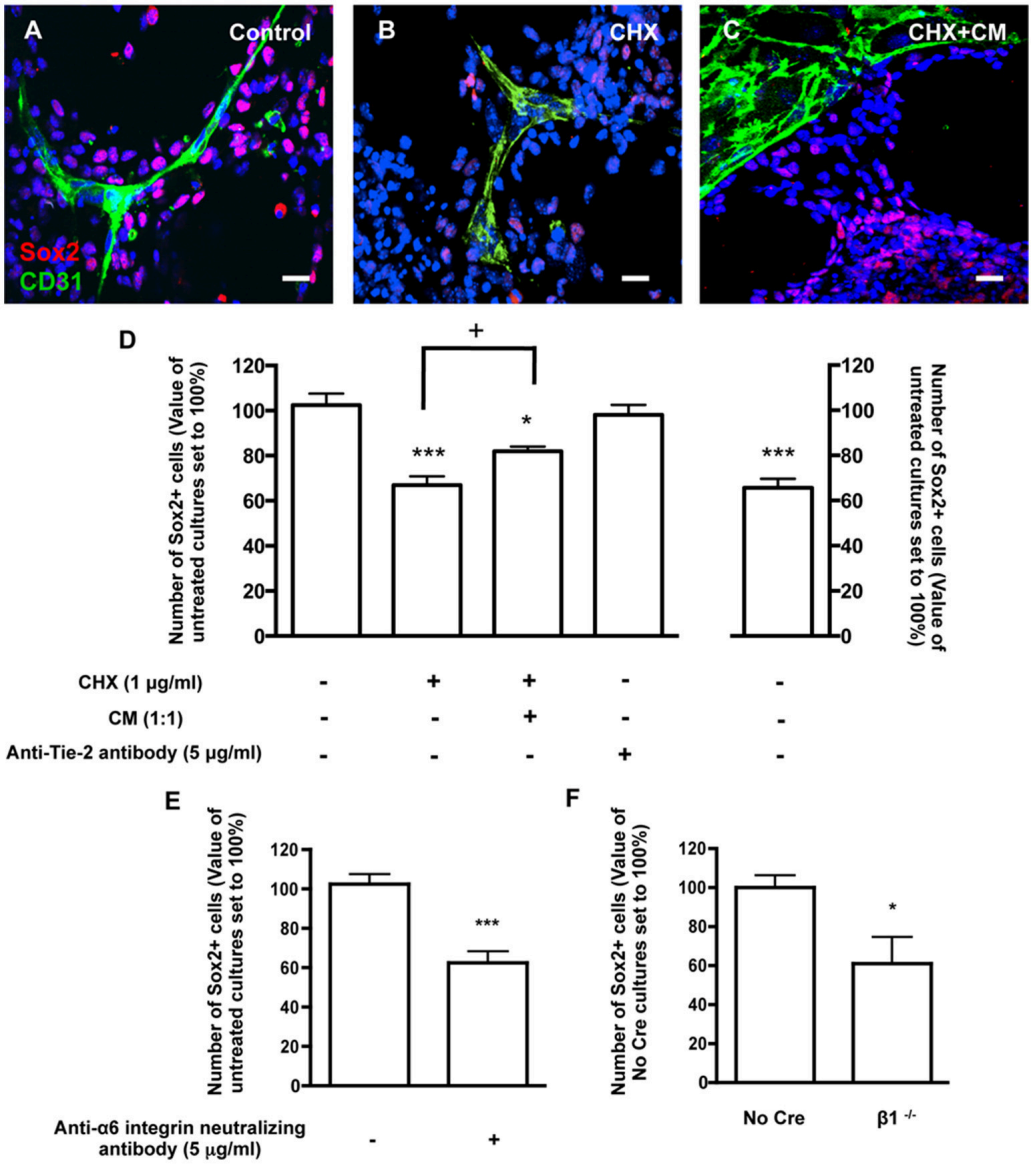
**Contacts between SVZ cells and BEC promote SVZ cell stemness. (A–C)** Representative confocal digital images depicting stem-like cells in BEC and SVZ cells cocultures (green staining for CD31, red staining for Sox2 and Hoechst 33342 staining in blue). **(A)** Control cocultures. **(B)** Cocultures with CHX-treated BEC, in SFM. **(C)** Cocultures with CHX-treated BEC, in SFM and BEC-CM (1:1). Scale bars, 20 μm. **(D)** Left: Bar graphs show the number of Sox2+ cells as a percentage of the total cells contacting BEC. Right: Graph displays the number of Sox2+ cells as a percentage of the total number of cells in the pseudomonolayer of differentiation, i.e., non-contacting BEC. Control values were normalized to 100%. ^*^P < 0.05, ^***^P < 0.001, using one-way ANOVA. + *P* < 0.05 using the unpaired *t*-test for comparison to cocultures with CHX-treated BEC. **(E,F)** α6β1 integrin mediates stemness induced by contact with BEC. Bar graph shows the number of Sox2+ nuclei as a percentage of the total cells contacting BEC in Control cocultures, in anti-α6 integrin neutralizing antibody-incubated cocultures (**E**, ^***^P < 0.001 using the unpaired *t*-test for comparison to Control cocultures) and in cocultures with β1^−/−^ SVZ cells (**F**, ^*^P < 0.05 using the unpaired *t*-test for comparison to No Cre cocultures).

To assess the involvement of direct contact between BEC and SVZ cells in stemness, cocultures were stained for Sox2, a stem/progenitor cell marker. Within the population of SVZ cells contacting BEC, 42.45 ± 2.19% were Sox2+ (3013 Hoechst-labeled SVZ cells in contact with BEC were counted). This number was normalized to 100% (Figures 4A,D). In contrast, the percent of Sox2+ cells in the SVZ population that were not in contact with BEC was significantly smaller (*P* < 0.001, 12652 Hoechst-labeled SVZ cells not contacting BEC were counted, Figure 4D), which indicates that contact with BEC plays a significant role in the maintenance of the SVZ stem cell state. Accordingly, the percentage of Sox2+ cells decreased within cells contacting CHX-treated BEC (*P* < 0.001, Figures 4B,D). Interestingly, incubation of cocultures with CHX-treated BEC with BEC-conditioned media partially restored the expression of Sox2 in SVZ cells contacting CHX-treated BEC (*P* < 0.05 as compared to cocultures with CHX-treated BEC, Figures 4C,D) demonstrating that BEC-derived soluble factors also contribute to stemness maintenance in SVZ cells. However, Ang-1 was not involved in this process as coculture with an anti-Tie2 receptor antibody did not modify the number of Sox2+ SVZ cells contacting BEC (96.38 ± 6.21%, Figure 4D). Cocultures were then performed in the presence of the anti-α6 neutralizing antibody. The number of Sox2+ SVZ cells contacting BEC decreased as compared to non-treated cocultures (*P* < 0.001, Figure 4E). Furthermore, the number of No Cre Sox2+ cells contacting BEC was 35.71 ± 6.21% (588 cells counted) and was similar to that obtained with WT SVZ cultures This number was normalized to 100%. The percentage of β1^−/−^ Sox2+ cells in contact with BEC was decreased (*P* < 0.05, Figure 4F). These effects were not due to a decrease in Sox2 expression in the β1^−/−^ cultures compared to No Cre cells as verified by Western blot (data not shown). Together, these data show that the close interaction between SVZ and BEC can modulate stemness in SVZ cells, and that this function is regulated through α6β1 integrin in SVZ cells.

### Contact with BEC did not affect SVZ neuronal differentiation

**Figure 5:**
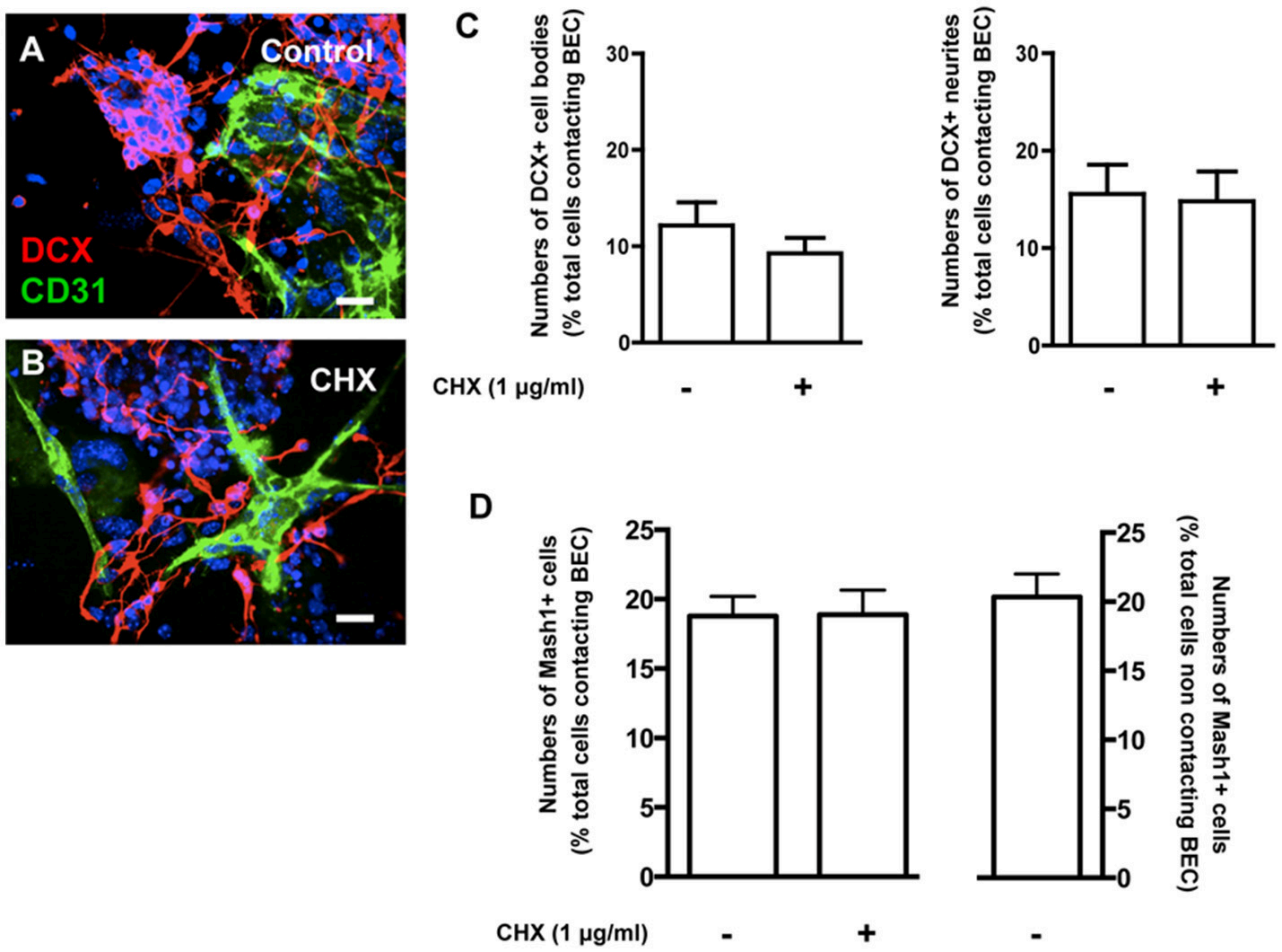
**Contacts between SVZ cells and BEC do not affect neuronal differentiation from SVZ cells. (A,B)** Representative confocal digital images depicting DCX+ cells in BEC and SVZ cells cocultures (green staining for CD31, an endothelial cell marker, red staining for DCX, a marker of migrating neuroblasts, and Hoechst 33342 staining in blue). **(A)** Control cocultures, **(B)** Cocultures with CHX-treated BEC. Scale bars, 20 μm. **(C,D)** Bar graph shows the number of DCX+ cell bodies (left portion of the graph) and neurites (right portion of the graph) **(C)** and Mash1+ cells **(D)** as a percentage of the total cells contacting BEC in Control and in CHX-treated cocultures.

Neuronal differentiation was evaluated based on staining for doublecortin (DCX) in untreated (Control) and CHX-treated BEC cocultures (Figures 5A–C). There were no differences in the number of DCX+ cell bodies or neurites contacting BEC in Control (1221 Hoechst-labeled SVZ cells contacting BEC were counted) and in CHX-treated BEC cocultures (1461 Hoechst-labeled SVZ cells contacting BEC were counted, Figure 5C). Similar results were obtained when analyzing SVZ progenitors labeled with Mash1, a neuronal transcription factor, further demonstrating that EC contacts did not affect neuronal commitment and differentiation (1938 Hoechst-labeled SVZ cells contacting BEC, 1563 Hoechst-labeled SVZ cells contacting CHX-treated BEC, and 4461 Hoechst-labeled SVZ cells that did not contact BEC, were counted, Figure 5D).

### Laminin regulates stemness through notch signaling and increased self-renewing divisions

**Figure 6:**
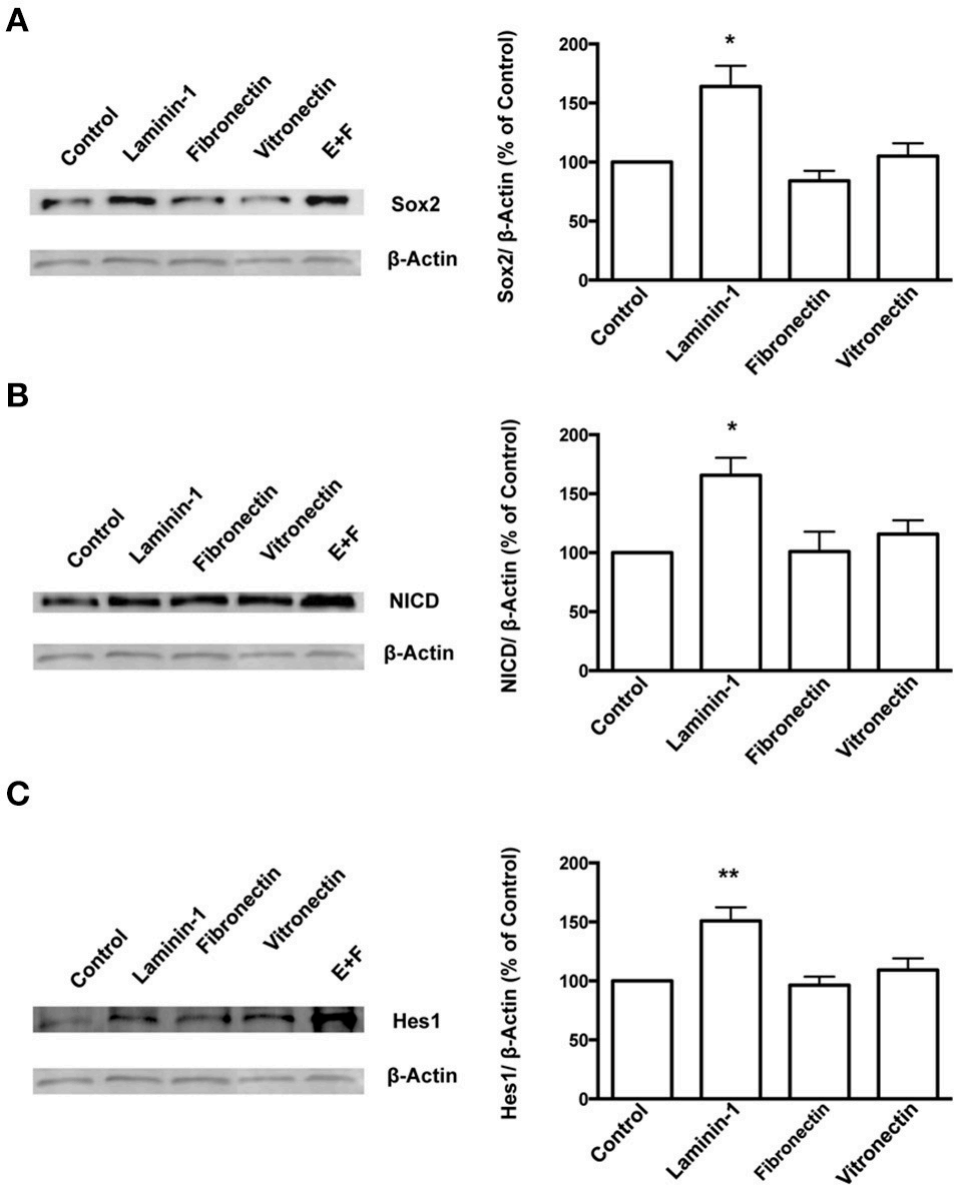
**Laminin-1 activates the Notch signaling pathway and sustains stemness in SVZ cells**. Western blots (left) and respective quantification (right) of protein levels of Sox2 **(A)**, NICD **(B)**, and Hes1 **(C)** in SVZ cells plated for 24 h on poly-D-lysine (control), laminin-1, fibronectin, or vitronectin. Bar graphs show the quantification of protein levels relative to levels obtained in cultures plated onto poly-D-lysine. Quantifications in free-floating neurospheres cultured in the presence of EGF and FGF-2 (E+F) were used as a positive control. ^*^P < 0.05 and ^**^P < 0.01 using one-way ANOVA for comparison to values obtained in cultures plated onto poly-D-lysine.

To further understand the role of the ECM protein laminin and α6β1 integrin in SVZ cells' stemness, SVZ cells were cultured for 72 h in SFM devoid of growth factors in culture dishes coated with poly-D-lysine alone or with either laminin-1 or other BEC-derived ECM molecules such as fibronectin or vitronectin. Levels of Sox2 were determined in cultures plated onto poly-D-lysine alone and set to 100%. Laminin-1, but not fibronectin nor vitronectin, induced an increase in Sox2 levels (poly-D-lysine vs. laminin-1: *P* < 0.05, Figure 6A). Levels of Sox2 protein in free-floating neurospheres cultures in the presence of EGF and FGF-2 were evaluated as positive controls as these conditions promote stemness in SVZ cells (Figure 6A). These results indicate that laminin-1 specifically sustains stemness in SVZ cells.

Studies have shown that the Notch signaling pathway regulates stem cell maintenance (Androutsellis-Theotokis et al., 2006; Aguirre et al., 2010). Therefore, levels of the Notch intracellular domain (NICD), the cleaved and activated form of Notch1 receptor, and of Hes1, a downstream effector of Notch, were quantified in SVZ cells cultured for 72 h on BEC-derived substrates as a measure of Notch activation. The results showed that laminin-1 triggers greater Notch activation as compared to the other ECM substrates tested (levels of NICD, poly-D-lysine vs. laminin-1: *P* < 0.05, Figure 6B; levels of Hes1: poly-D-lysine vs. laminin-1: *P* < 0.01, Figure 6C).

**Figure 7:**
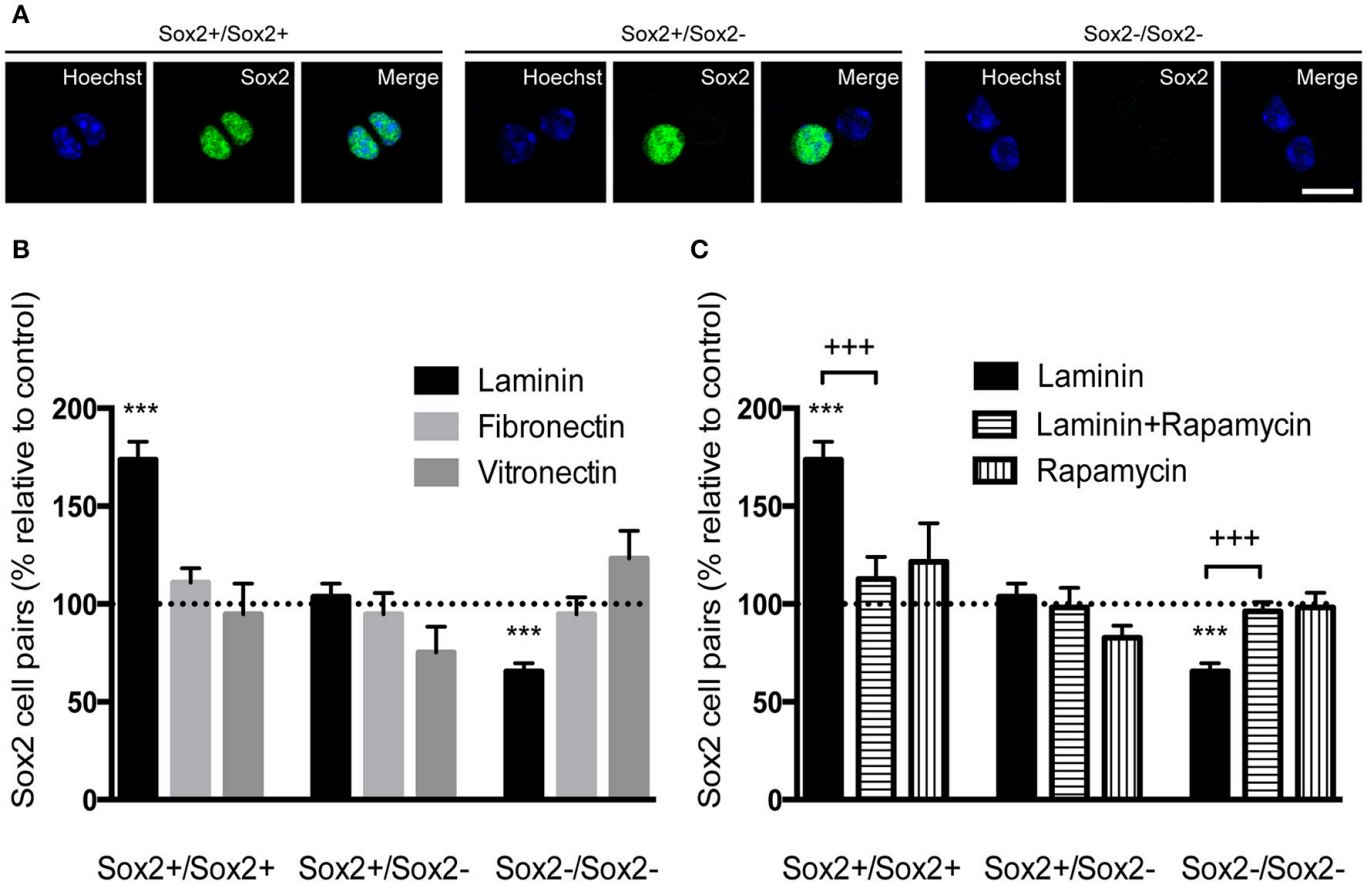
**Laminin-1 promotes self-renewing divisions of SVZ precursors via the mTOR pathway. (A)** Confocal digital images of cell pairs obtained following (left) the symmetrical division of a SVZ cell into 2 Sox2+ cells, (middle) the asymmetrical division into a Sox2+ and a Sox2− progenitor and (right) the symmetrical terminal division into 2 Sox2− progenitors. Scale bars, 10 μm. **(B)** Bar graph indicates the percentage of Sox2+/Sox2+, Sox2+/Sox2−, and Sox2−/Sox2− cell pairs in culture when cells were plated on laminin-1, fibronectin or vitronectin, relative to control condition (poly-D-lysine, set at 100%). Note that laminin-1 promotes self-renewal by increasing the number of divisions in Sox2+/Sox2+ pairs at the expense of terminal divisions. **(C)** Bar graph shows the percentage of Sox2+/Sox2+, Sox2+/Sox2−, and Sox2−/Sox2− cell pairs relative to control condition (poly-D-lysine, set at 100%) obtained from cells plated on laminin-1, and in the absence or the presence of 20 nM of the mTOR inhibitor rapamycin. Data shows that mTOR is important in the induction of self-renewal by laminin-1. ^***^P < 0.001, ^+++^*P* < 0.001 using unpaired *t*-test.

The capacity of self-renewal is a central feature of the stem cell state and is defined by the possibility of stem cells to divide and generate two daughter cells that are identical to the “mother” stem cell or a stem cell and a progenitor cell. However, there is loss of self-renewal capacity when a stem cell terminally divides into two progenitor cells. The capacity of laminin-1, compared to other ECM molecules, to favor self-renewing divisions was determined on SVZ single cells plated for 24 h and stained for Sox2. Cell pairs either Sox2+/Sox2+, Sox2+/Sox2− or Sox2−/Sox2− were identified (Figure 7A). Numbers of cell pairs were determined in each condition and compared to numbers obtained on poly-D-lysine set to 100% (corresponding to a total of 400 cell pairs counted). Only cells grown on laminin-1 increased self-renewing divisions (Sox2+/Sox2+) as compared to poly-D-lysine (poly-D-lysine vs. laminin-1: *P* < 0.001). Consistent with the idea that laminin-1 predominantly induces self-renewal, laminin-1 significantly decreased the number of differentiating divisions (Sox2−/Sox2−) (poly-D-lysine vs. laminin-1: *P* < 0.001; Figure 7B).

It has been shown that Notch receptor activation leads to the expression of Hes genes through cytoplasmatic intermediate mediators including the serine-threonine kinase mTOR (Androutsellis-Theotokis et al., 2006). To disclose whether mTOR mediated laminin-induced self-renewing divisions on SVZ precursors, cell pair assays were performed in the presence of 20 nM of the mTOR inhibitor rapamycin. While laminin-1 increased the numbers of Sox2+/Sox2+ pairs (poly-D-lysine vs. laminin-1: *P* < 0.001) at the expense of Sox2−/Sox2− (*P* < 0.001), these effects were inhibited in the presence of rapamycin (*P* < 0.001; Figure 7C) (Numbers of cell pairs counted were compared to numbers obtained on poly-D-lysine set to 100%, corresponding to a total number of 763 cell pairs counted). Taken together, these results suggest that laminin sustains stemness *via* activation of the Notch and mTOR signaling pathways.

## Discussion

This work was undertaken to determine whether physical interaction between SVZ stem/progenitor cells and BEC modulate stem cell properties. Using cocultures of primary BEC with SVZ neurospheres, we demonstrated that physical contact with BEC promoted SVZ cell proliferation and maintained Sox2 expression. These effects were exerted, at least partially, through the binding of BEC-derived laminin to α6β1 integrin, and subsequent Notch and mTOR signaling pathways.

Cellular interactions with blood vessels have been described to modulate SVZ stem cells properties (Shen et al., 2008; Tavazoie et al., 2008; Snapyan et al., 2009; Kojima et al., 2010; Kokovay et al., 2010). Using cocultures, we found that proliferation and Sox2 expression are increased in SVZ cells in contact with BEC. However, these effects may also be due to diffusible soluble factors, based on the fact that NSCs derived from embryonic and postnatal rodents remain undifferentiated, and proliferate in the presence of EC-derived diffusible cues (Shen et al., 2004; Gama Sosa et al., 2007; Plane et al., 2010; Sun et al., 2010). Therefore, we also determined the proportion of Sox2+ cells and BrdU+ cells in SVZ cells that were not in contact with BEC, but were exposed to EC diffusible factors. The proportions of Sox2+ and BrdU+ cells were lower as compared to those obtained when SVZ cells contact BEC demonstrating that physical interaction play a major role in the observed effects. Moreover, we used CHX to inhibit the turnover of contact proteins in BEC and found that proliferation and Sox2 expression in SVZ cells were reduced to levels similar to those obtained in SVZ cells that were not in contact with BEC. As CHX action is not specific and might inhibit both the synthesis of contact proteins and of EC-derived soluble factors, cocultures were performed in which EC soluble factors were restored by incubation with BEC SFM-conditioned medium. Only partial recovery of the effects was observed further emphasizing the importance of physical contacts. Consistent with our findings, Mathieu and collaborators performed cocultures of spheres from embryonic mice forebrain and murine EC lines and found that EC sustained expression of Sox2 and Nestin (Mathieu et al., 2006). The role of EC supporting the expansion of stem cells was also reported for gliomas (Borovski et al., 2009; Galan-Moya et al., 2011; Zhu et al., 2011).

In the present study, we found no evidence of BEC inducing neuronal differentiation. In contrast, direct coculture of mouse or human embryonic NSCs with EC increased neuronal differentiation (Mathieu et al., 2006; Gama Sosa et al., 2007; Chintawar et al., 2009). Although there are differences in the developmental status of the cells used in these studies as compared to our cells, the timing of the cocultures may be critical in explaining the apparent discrepancies: indeed we examined neuronal differentiation and commitment at 24 h due to the viability of BEC, while the other studies used later time points (up to 8 days). Nevertheless, we did see a tendency for the number of DCX+ cells to increase when SVZ cells were grown on laminin-1 for 72 h (Supplementary Figure 2), which correlates well with previous studies (Flanagan et al., 2006; Mruthyunjaya et al., 2010).

SVZ progenitors where shown to adhere to laminin-rich BEC-derived ECM *via* α6β1 integrins (Shen et al., 2008; Kazanis et al., 2010). We showed that BEC and Nestin+ SVZ cells expressed laminin and α6β1 integrin, respectively, and that laminin-integrin interaction sustained proliferation and Sox2 expression in SVZ cells. Strikingly, a recent work suggests that direct cell–cell interactions with endothelial cells reduce SVZ cell proliferation (Ottone et al., 2014). The difference in the source of endothelial cells used may explain this discrepancy. We used primary endothelial cells obtained from whole adult mice brains rather than the bEnd.3.1 mouse brain endothelial cell line. It is established that endothelial-derived diffusible factors have different neurogenic properties according to endothelial cells localization within the brain (Crouch et al., 2015). Therefore endothelial cells may present different contact molecules at their surface according to their origin. In the study of Ottone et al. (2014) endothelial to NSCs contacts are mediated by endothelial ephrinB2 and Jagged1 and result in reduced proliferation by dampening of the MAPK pathway. Here, we presented evidence of a pro-proliferative effect of heterocellular contacts through endothelial secreted laminin and α6β1 integrin. Nevertheless, in both studies, contact with endothelial cells promotes stemness capacities of SVZ cells.

Expression of α6β1 integrins is a hallmark of stem cells including from non-neural tissue (Watt, 1998; Shinohara et al., 1999; Ramalho-Santos et al., 2002; Hall et al., 2006; Yovchev et al., 2008; Lathia et al., 2010; Notta et al., 2011). α6β1 integrin expression correlates with stem cell properties. Indeed, during cortical development, enhanced integrin β1 signaling in the chick neuroepithelium increases the expansion of Sox2+ cells and inhibits their differentiation (Long et al., 2016). In mice, β1-dependent anchoring of radial glia NSCs to laminin-rich ventricular surface regulates interkinetic nuclear migration and division orientation of NSC, two parameters necessary for proper cortical lamination (Belvindrah et al., 2007; Loulier et al., 2009). Accordingly, perturbations in corticogenesis were observed in α6 integrin –/– mice (Georges-Labouesse et al., 1998). Specific downregulation of α6 integrin expression decreased self-renewal and tumor formation capacities of glioma cancer stem cells (Lathia et al., 2010).

Regarding SVZ cells, β1 integrin positively regulates proliferation and stemness maintenance through activation of the MAPK signaling pathway (Campos et al., 2004; Leone et al., 2005). Furthermore, β1 integrin signaling inhibits astroglial differentiation in SVZ cells cultures (Pan et al., 2014). There is a strong correlation between the expression of α6β1 integrin and the proliferative status of stem/progenitor cells. In the SVZ *in vivo*, β1 integrin is not expressed by quiescent B cells but in transient amplifying cells and neuroblasts (Kazanis et al., 2010). Stromal cell-derived factor-1 (SDF-1) secreted by EC upregulates the expression of α6 integrin in C cells to promote their adhesion to laminin (Kokovay et al., 2010). *In vivo*, adhesion to ECM components promotes exposure to extracellular cues and crosstalk with integrin signaling (Hynes, 2002, 2009; ffrench-Constant and Colognato, 2004). We have provided here a demonstration that laminin-integrin binding is by itself enough to regulate SVZ cell properties. This is consistent with studies in the pancreas where blood vessel-derived laminin interacts with α6β1 integrin of pancreatic β-cells to promote their proliferation (Nikolova et al., 2006).

Laminin maintains SVZ functions *via* activation of α6β1 integrins. α6β1 integrins bind laminin α5β1γ1, laminin α3β3γ2, laminin α1β1γ1 and laminin α4β1γ1 with higher affinity for laminin α5β1γ1 (for review see Barczyk et al., 2010). In the basal lamina of blood vessels and fractones in the SVZ, laminin subunits α1,2,5 and β1 have been detected (Shen et al., 2008; Kazanis et al., 2010). *In vitro*, laminin coating is used to culture stem cells. DG cells from neonatal mice and embryonic human and mouse cortices retained stem/progenitor cell capacities when plated on laminin (Flanagan et al., 2006; Imbeault et al., 2009). Protocols described successful propagation of stem/progenitor cells from SVZ and glioma in monolayers by using laminin coating (Pollard et al., 2006, 2009). Soluble laminin added to the culture medium increased the proliferation rate of human embryonic cortex cells in a β1 integrin-dependent manner (Hall et al., 2008). The Notch signaling pathway is activated in SVZ cells plated on laminin-1 as shown by increased expression of NICD and Hes1. This pathway is crucial for the regulation of neural stem cell numbers (Androutsellis-Theotokis et al., 2006; Aguirre et al., 2010; Imayoshi et al., 2010; Basak et al., 2012). Notch signaling blockade by γ-secretase inhibitors reduced proliferation of glioma cancer stem cells while decreasing Hes1 mRNA levels (Fan et al., 2010). EC also sustained self-renewal and proliferation capacities of glioblastoma tumor stem cells, and specific elimination of EC decreased mRNA levels of Notch effectors in tumor cells, demonstrating that EC-derived paracrine factors, including contact factors, promote stemness *via* activation of the Notch pathway on these cells (Hovinga et al., 2010). Our study shows a crosstalk between integrin and the Notch pathway. Such interaction has been demonstrated in neurospheres from newborn mice where β1 integrin activation increases NICD translocation to the nucleus (Campos et al., 2006). Although endothelial cells may provide Notch ligands that activate Notch pathway and self-renewal in glioma cancer stem cells (Zhu et al., 2011), our results demonstrate that laminin secretion by endothelial cells is sufficient to activate Notch1 in SVZ cells. However, autocrine/paracrine secretion of Notch ligands following integrin activation cannot be excluded. Indeed, in endothelial cells, α6β1 integrin activation triggers an increase in Delta-like 4 ligand expression and subsequent Notch1 cleavage to NICD (Estrach et al., 2011). Moreover, besides the Notch pathway, other intracellular mediators of integrin activity such as Id proteins have been identified to regulate stemness in the vascular niche (Niola et al., 2012). We also show that the mTOR kinase mediates self-renewal in stem cells cultured on laminin-1 by counting less numbers of Sox2+/+ cell pairs under inhibition of this pathway. The mTOR kinase is required for the survival and maintenance of the stem cell state in NSCs and glioma cancer stem cells (Sato et al., 2010; Galan-Moya et al., 2011). Moreover, mTOR is a mediator of the Notch-Hes pathway in NSCs (Androutsellis-Theotokis et al., 2006).

In this study, we provide evidence that laminin modulates SVZ cell division and favors self-renewing divisions suggesting that laminin orients cell divisions and influences cell fate decisions. It has been shown that laminin and integrin α6β1 regulate asymmetric divisions of NSCs in the ventricular zone during neocortical development (Lathia et al., 2007; Loulier et al., 2009). Subcellular mechanisms regulating cell polarity and fate specification of progenitors during corticogenesis ensure the appropriate orientation of the mitotic spindle and the asymmetric inheritance of the mother cell centrosome (Götz and Huttner, 2005; Wang et al., 2009). It is tempting to speculate that integrin signaling in the SVZ triggered by BEC-derived laminin may interfere with these polarity mechanisms. In line with this, laminin directs centrosome positioning and polarization of granule cell precursors during postnatal cerebellum development *via* α6β1 integrins activation (Gupta et al., 2010).

We demonstrate that heterocellular interactions between BEC and SVZ cells enhance proliferation and self-renewal properties of SVZ cells. Our studies further underscore the importance of BEC-derived ECM components and integrin signaling in regulating stem cells and foresee that manipulation of these molecular targets may prove to be useful in NSCs-based regenerative therapies.

## Author contributions

AR: design of experiments, acquisition of data, analysis and interpretation of data, writing the manuscript; SG: acquisition of data, analysis and interpretation of data, revising the manuscript; SS: acquisition of data, analysis and interpretation of data, revising the manuscript; LB: acquisition of data, analysis and interpretation of data, revising the manuscript; TC: providing reagents and funding, revising the manuscript; JR: providing reagents and funding, revising the manuscript; FH: providing reagents and funding, design of experiments, revising the manuscript; FA: providing reagents and funding, conception of study, design of experiments, acquisition of data, analysis and interpretation of data, revising the manuscript.

## Funding

This work was supported by FCT Portugal and by FEDER, PTDC/SAU-NEU/101783/2008, PTDC/SAU-NEU/104415/2008, SFRH/BD/32944/2006, PTDC/BIA-BCN/112730/2009 (Glial Cell biology lab), Grant n° 96542 from the Calouste Gulbenkian foundation, Fondation pour la Recherche Médicale (FRM, équipe labellisée, S.H.).

### Conflict of interest statement

The authors declare that the research was conducted in the absence of any commercial or financial relationships that could be construed as a potential conflict of interest.

## Supplementary material

Supplementary Figure 1 **BEC are viable for 24 h, but not 48 h, in SFM medium. (A)** Bar graph depicts the cell viability of BEC, determined by MTT assay, at 24 h after incubation in normal BEC media (Control) and in serum free media (SFM). **(B)** Bar graph depicts the cell viability of BEC, determined by MTT assay, at 48 h after incubation in Control and in SFM, ^***^*P* < 0.001, using the unpaired Student *t*-test. **(C)** Bar graph depicts the cell death of BEC, determined by TUNEL assay, at 24 h after incubation in Control and in SFM plus SFM conditioned by SVZ cells (CM) (1:1). **(D)** Bar graph depicts the cell death of BEC, determined by TUNEL assay, at 48 h after incubation in Control and in SFM plus SFM conditioned by SVZ cells (CM) (1:1). ^***^*P* < 0.001, using the unpaired Student *t*-test.

Supplementary Figure 2 **Laminin-1 tends to increase neuronal, but not astroglial, differentiation of SVZ**. Differentiation of SVZ cells plated on poly-D-lysine, laminin-1, fibronectin and vitronectin for 72 h. Bar graphs show the percentage of DCX positive (+) neuroblasts and GFAP+ astrocytes in each condition. No significant differences were obtained using a two-way ANOVA with Tukey's multiple comparisons test.

Supplementary Table 1 **Information relative to the primary antibodies used in immunochemistry**.

Supplementary Table 2 **Information relative to the primary antibodies used in Western blot**.

Supplementary Table 3 **Information relative to the secondary antibodies used in western blot**.
