# Supplementary figures and images for: Heterocellular Contacts with Mouse Brain Endothelial Cells Via Laminin and α6β1 Integrin Sustain Subventricular Zone (SVZ) Stem/Progenitor Cells Properties
Source: Front Cell Neurosci. 2016 Dec 15;10:284. doi: 10.3389/fncel.2016.00284 (PMC5156690; doi:10.3389/fncel.2016.00284)

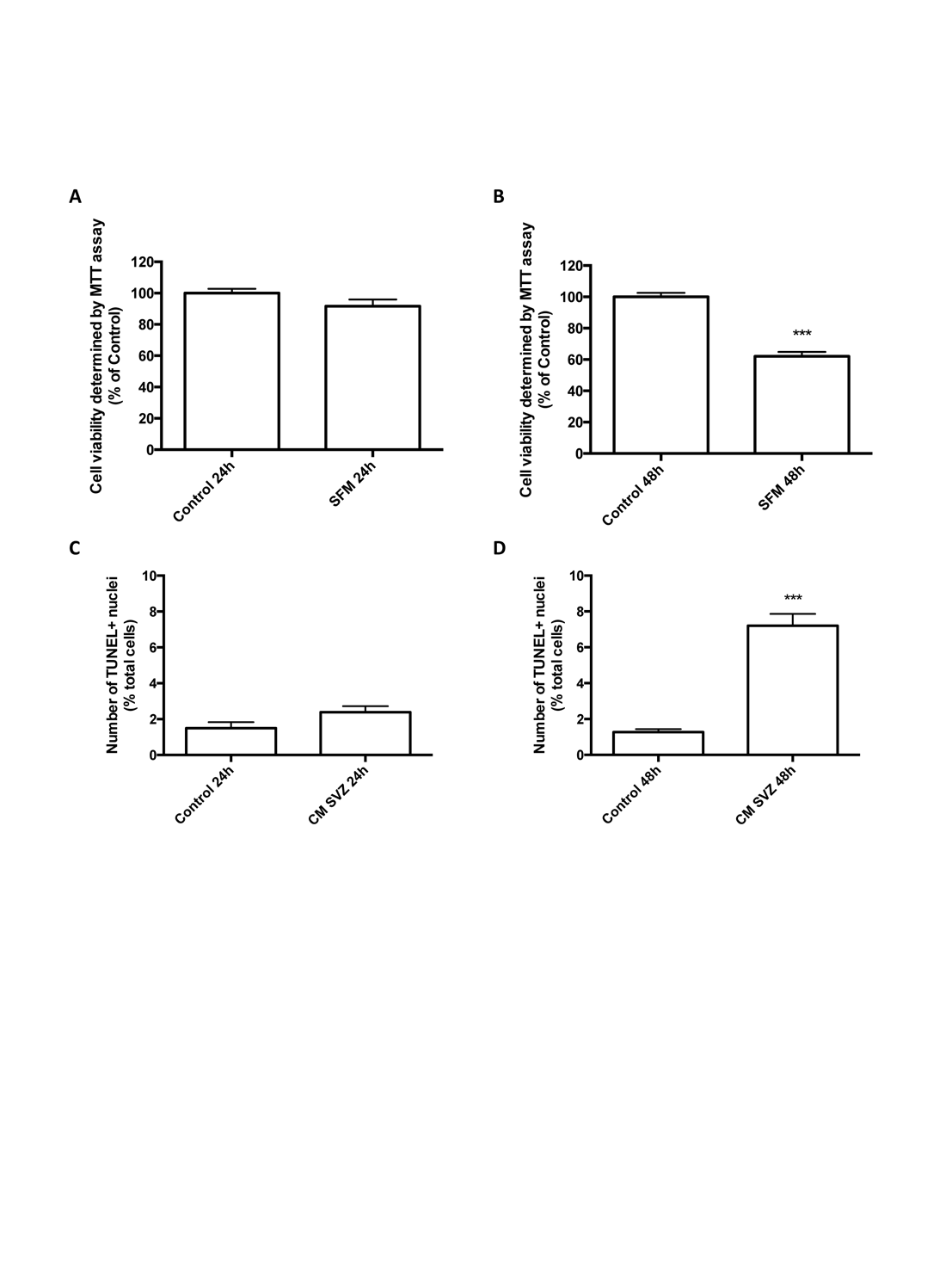

Supplement: Supplementary Figure 1 — BEC are viable for 24 h, but not 48 h, in SFM medium. (A) Bar graph depicts the cell viability of BEC, determined by MTT assay, at 24 h after incubation in normal BEC media (Control) and in serum free media (SFM). (B) Bar graph depicts the cell viability of BEC, determined by MTT assay, at 48 h after incubation in Control and in SFM, ***P < 0.001, using the unpaired Student t-test. (C) Bar graph depicts the cell death of BEC, determined by TUNEL assay, at 24 h after incubation in Control and in SFM plus SFM conditioned by SVZ cells (CM) (1:1). (D) Bar graph depicts the cell death of BEC, determined by TUNEL assay, at 48 h after incubation in Control and in SFM plus SFM conditioned by SVZ cells (CM) (1:1). ***P < 0.001, using the unpaired Student t-test. [file Image1.TIF]

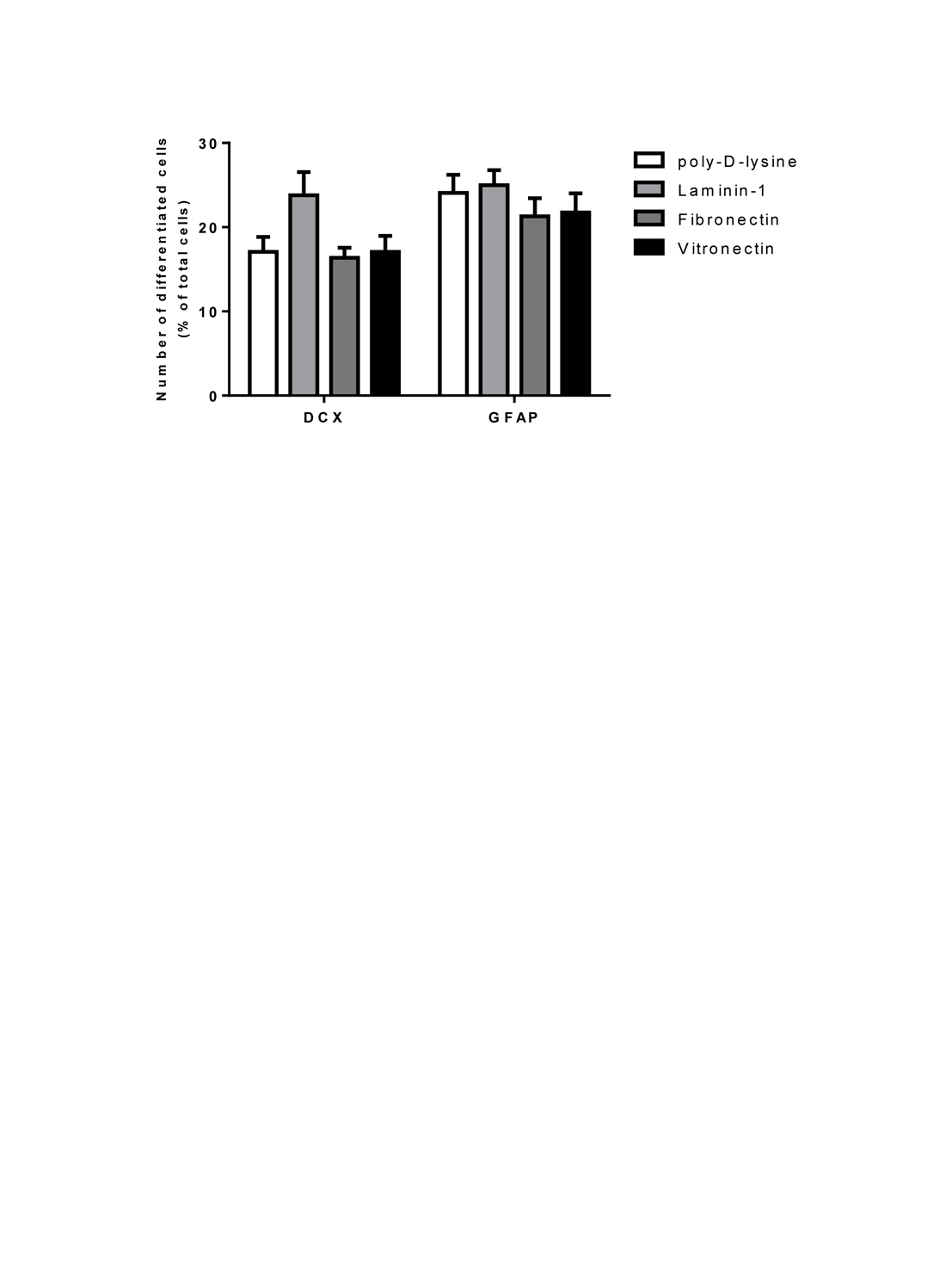

Supplement: Supplementary Figure 2 — Laminin-1 tends to increase neuronal, but not astroglial, differentiation of SVZ. Differentiation of SVZ cells plated on poly-D-lysine, laminin-1, fibronectin and vitronectin for 72 h. Bar graphs show the percentage of DCX positive (+) neuroblasts and GFAP+ astrocytes in each condition. No significant differences were obtained using a two-way ANOVA with Tukey's multiple comparisons test. [file Image2.TIF]
