# Supplementary material for: Heterocellular Contacts with Mouse Brain Endothelial Cells Via Laminin and α6β1 Integrin Sustain Subventricular Zone (SVZ) Stem/Progenitor Cells Properties
Source: Front Cell Neurosci. 2016 Dec 15;10:284. doi: 10.3389/fncel.2016.00284 (PMC5156690; doi:10.3389/fncel.2016.00284)
Supplement: Supplementary Table 1 — Information relative to the primary antibodies used in immunochemistry. [file Table1.DOC]

Supplementary Table 1: Information relative to the primary antibodies used in immunochemistry.

| **Antigen** | **Company** | **Catalog number** | **Source** | **Dilution** |
| --- | --- | --- | --- | --- |
| **BrdU** | Invitrogen | A21304 | Mouse | 1/100 |
| **CD31** | BD Biosciences | 550274 | Rat | 1/200 |
| **DCX** | Santa Cruz Biotechnology (Santa Cruz, CA, USA) | Sc-8066 | Goat | 1/200 |
| **Doublecortin (DCX)** | Cell Signaling Technology Inc. (Beverly, MA, USA) | 4604 | Rabbit | 1/200 |
| **Glial fibrillary acidic protein (GFAP)** | Cell Signaling Technology Inc. | 3670 | Mouse | 1/200 |
| **GFAP** | Sigma-Aldrich | G9269 | Rabbit | 1/500 |
| **Laminin-1 (α1, β1,γ1) and laminin-2 (α2, β1,γ1)** | Abcam (Cambridge, UK) | Ab7463 | Rabbit | 1/100 |
| **Mash1** | BD Biosciences (Franklin Lakes, NJ USA) | 556604 | Mouse | 1/50 |
| **Nestin** | Abcam | Ab6142 | Mouse | 1/200 |
| **Olig2** | Millipore (Billerica, MA, USA) | AB9610 | Rabbit | 1/200 |
| **Sox2** | Santa Cruz Biotechnology | Sc-17320 | Goat | 1/500 |
| **α6 integrin** | R&D Systems (Minneapolis, MN, USA) | MAB13501 | Rat | 1/100 |
| **α6 integrin** | Millipore | MAB1378 | Rat | 1/100 |
| **β1 integrin** | Abcam | Ab52971 | Rabbit | 1/100 |
|  |  |  |  |  |
