# Supplementary material for: Heterocellular Contacts with Mouse Brain Endothelial Cells Via Laminin and α6β1 Integrin Sustain Subventricular Zone (SVZ) Stem/Progenitor Cells Properties
Source: Front Cell Neurosci. 2016 Dec 15;10:284. doi: 10.3389/fncel.2016.00284 (PMC5156690; doi:10.3389/fncel.2016.00284)
Supplement: Supplementary Table 2 — Information relative to the primary antibodies used in Western blot. [file Table2.DOCX]

Supplementary Table 2: Information relative to the primary antibodies used in Western blot.

| **Antigen** | **Company** | **Catalog number** | **Source** | **Loaded protein (μg)** | **Dilution** |
| --- | --- | --- | --- | --- | --- |
| **α6 integrin** | R&D Systems | MAB13501 | Rat | 50 | 1/100 |
| **β-actin** | Sigma-Aldrich | A1978 | Mouse | Loading control | 1/2000 |
| **β1 integrin** | Invitrogen | 44-870G | Rabbit | 70 | 1/1000 |
| **GAPDH** | Santa Cruz Biotechnology | Sc-25778 | Rabbit | Loading control | 1/10000 |
| **Hes1** | Millipore | AB5702 | Rabbit | 30 | 1/1000 |
| **Laminin (α1, β1,γ1) and laminin-2 (α2, β1,γ1)** | Abcam | Ab7463 | Rabbit | 25 | 1/100 |
| **Notch intracellular domain (NICD)** | Abcam | ab27526 | Rabbit | 50 | 1/100 |
| **Sox2** | Santa Cruz Biotechnology | Sc-17320 | Goat | 50 | 1/100 |
