# Supplementary material for: Heterocellular Contacts with Mouse Brain Endothelial Cells Via Laminin and α6β1 Integrin Sustain Subventricular Zone (SVZ) Stem/Progenitor Cells Properties
Source: Front Cell Neurosci. 2016 Dec 15;10:284. doi: 10.3389/fncel.2016.00284 (PMC5156690; doi:10.3389/fncel.2016.00284)
Supplement: Supplementary Table 3 — Information relative to the secondary antibodies used in western blot. [file Table3.DOCX]

Supplementary Table 3: Information relative to the secondary antibodies used in western blot.

| **Secondary antibody** | **Company** | **Catalog number** | **Dilution** |
| --- | --- | --- | --- |
| **Alkakine phosphatase-conjugated Goat anti-Rabbit** | GE Healthcare (Buckinghamshire, UK) | RPN 5783 | 1/20000 |
| **Alkakine phosphatase-conjugated Goat anti-Mouse** | GE Healthcare | RPN5781 | 1/20000 |
| **Alkakine phosphatase-conjugated Rabbit anti-Goat** | Sigma-Aldrich | A4187 | 1/5000 |
| **IRDye® 680-conjugated Goat anti-rabbit** | Licor Biosciences (Lincoln, Nebraska, USA) | 926-32221 | 1/5000 |
| **IRDye® 680-conjugated Goat anti-rat** | Licor Biosciences | 926-32229 | 1/5000 |
